# Microglial and neuronal subpopulations associated with cognitive resilience to amyloid deposition in genetically diverse mice

**DOI:** 10.1002/alz.093491

**Published:** 2025-01-03

**Authors:** Asli Uyar, Sarah E Heuer, Daniel A Skelly, Gregory W Carter, Erik B Bloss, Gareth R Howell

**Affiliations:** ^1^ The Jackson Laboratory, Bar Harbor, ME USA; ^2^ Ann Romney Center for Neurologic Diseases, Department of Neurology, Brigham and Women’s Hospital and Harvard Medical School, Boston, MA USA

## Abstract

**Background:**

Data from human and model organism studies suggest that genetic background influences susceptibility and resilience to Alzheimer’s Disease (AD) neuropathology. We previously showed that, wild‐derived PWK/PhJ (PWK) mice carrying the APP/PS1 transgene (PWK.APP/PS1) exhibit cognitive and synaptic resilience compared to traditionally‐studied B6.APP/PS1 inbred mice in presence of amyloid beta (Aβ) plaque deposition. PWK.APP/PS1 mice also contain different proportions of transcriptionally‐defined microglia compared to B6.APP/PS1. The precise molecular mechanisms underlying these differences between strains remain unknown. In this study, we aim to identify and characterize cell populations in the hippocampi of genetically distinct B6 and PWK mouse strains, and investigate cell‐type specific gene expression patterns associated with cognitive resilience in presence of amyloid deposition.

**Method:**

We generated cohorts of B6.*APP/PS1* and PWK.*APP/PS1* transgenic female mice and wild‐type (WT) littermates as controls (n = 4 per strain/genotype group). At 8 months of age mice were euthanized, hippocampi collected, and single nuclear RNA sequencing (snRNAseq) was performed using 10x Genomics Chromium platform. Seurat R package (version 5.0.1) was used for major data analysis.

**Result:**

We identified subpopulations of microglial cells characterized by varying expression patterns of a set of genes including Hk2, Itgam and Dock8. Neuronal subtypes were clustered by top marker genes Plk5 and Adarb2. Oligodendrocytes, oligodendrocyte progenitor cells, and endothelial cells were marked by expression of Mog, Neu4 and Abcc9, respectively. Differential expression and pathway enrichment analysis revealed strain‐specific immune and cell signaling pathways altered in distinct cell populations in response to amyloid pathogenesis. Cross‐species mapping of these transcriptomic signatures with human hippocampal gene modules identified AD‐relevant molecular mechanisms observed in mouse models of AD at the cell‐type level.

**Conclusion:**

This study suggests that cell‐type specific transcriptomic response to amyloid is modulated by genetic background and our findings re‐iterate the importance of incorporating genetic diversity to model phenotypic and molecular heterogeneity in AD. Since the nuclei enriched in this dataset are enriched for neurons, future interrogations will provide evidence for specific neuronal populations and molecular mechanisms that differentiate cognitively resilient PWK from susceptible B6 mice, providing insights into mechanisms that can be leveraged to promote AD resilience.

